# TWO DECADES EVALUATING EXERCISE TREATMENTS FOR ALZHEIMER'S DISEASE: THE ANECDOTES OF AN EVIDENCE-BASED PATH

**DOI:** 10.1093/geroni/igac059.838

**Published:** 2022-12-20

**Authors:** Patricia Heyn

**Affiliations:** Center for Optimal Aging, Marymount University, Fairfax Station, Virginia, United States

## Abstract

The last 20 years have produced accumulative evidence supporting the benefits of regular exercise to improve cognition in older adults with cognitive impairments (OAwCIs). Thus, multiple systematic reviews, including meta-analyses, have contributed to the current literature. Although the field advanced significantly our understanding of the role of exercise on AD, still there is limited information on the overall prescription effectiveness. Thus, a novel overview review to evaluate the available meta-analysis studies from the randomized exercise trials for OAwCI will help in synthesizing the best evidence to generate prescription precision. Dr. Heyn will discuss her path, her studies, and the future of AD research and exercise prescription.

